# Pediatric Medication Prescribing Across Urgent Care Visits: An Epidemiologic View from a Primary Care Setting in the Kingdom of Saudi Arabia

**DOI:** 10.3390/medsci13030177

**Published:** 2025-09-05

**Authors:** Reem S. AlOmar, Nouf A. AlShamlan, Ahmed A. Al Yateem, Abdulrahman A. Al-Abdulazeem, Ahmed M. Al-Turki, Reema J. Alghamdi, Najla A. Alhamed, Sameerah Motabgani, Adam F. Aldhawyan, Malak A. Al Shammari

**Affiliations:** 1 Department of Family and Community Medicine, College of Medicine, Imam Abdulrahman Bin Faisal University, Dammam 31451, Saudi Arabia; 2 College of Medicine, Imam Abdulrahman Bin Faisal University, Dammam 31441, Saudi Arabia; 3 Pharmaceutical Affairs, King Fahad Specialist Hospital, Eastern Health Cluster, Dammam 32253, Saudi Arabia

**Keywords:** epidemiology, public health, urgent care, primary care, pediatrics, prescriptions, antibiotics

## Abstract

**Background:** Urgent care clinics (UCCs) embedded within primary healthcare settings play a vital role in managing acute, non-life-threatening conditions in children. However, limited data exist on medication prescribing patterns in such settings in the Kingdom of Saudi Arabia (KSA), particularly regarding antibiotic use. This study aimed to describe the epidemiology of pediatric urgent care visits and identify factors associated with prescribing within a model primary healthcare (PHC) center. **Methods:** A retrospective chart review was conducted for all urgent care visits made by pediatric patients (<14 years) at a model PHC center in the KSA for all visits in 2024. Sociodemographic variables, visit timing, diagnosis, and prescription data were extracted from electronic health records. Multivariable logistic regression was used to analyze predictors of medication prescribing. **Results:** Of the 1016 pediatric urgent care visits, 62.5% resulted in medication prescriptions, and 23.62% of those visits included at least one antibiotic, primarily penicillins (71.33%). Cephalosporins and tetracyclines were not prescribed. Prescriptions were 67% more likely among adolescents and 70% less likely among infants when compared to school-aged children (95% CI = 1.04–2.67 and 95% CI = 0.15–0.61, respectively). Respiratory and ENT-related diagnoses accounted for most prescriptions. No significant sex-based differences in prescribing were observed. **Conclusions:** The epidemiological patterns observed indicate that respiratory and ENT conditions, as well as seasonal peaks in autumn and winter, are the main drivers of prescribing in pediatric urgent care. These findings have implications for strengthening disease surveillance, anticipating service demand, guiding preventive interventions such as vaccination and health education, and supporting evidence-based planning of primary care resources.

## 1. Introduction

Urgent care clinics (UCCs) are healthcare clinics designed to provide timely outpatient medical services for acute, non-life-threatening conditions that require prompt attention but do not necessitate emergency department (ED) visits. According to the American Academy of Urgent Care Medicine, such clinics aim to fill the care gap between emergency services and scheduled primary care appointments, offering walk-in access to patients with conditions such as minor infections, injuries, or exacerbations of chronic illness [1]. Their value lies in decongesting EDs and increasing healthcare system responsiveness while maintaining cost-effectiveness [2].

Within primary care frameworks, urgent care plays a vital role, particularly when overseen by family physicians trained to manage a wide range of acute and chronic conditions. This comprehensive, patient-centered approach helps ensure appropriate triaging, especially in settings where urgent care is integrated into model family medicine centers [3].

In the Kingdom of Saudi Arabia (KSA), urgent care services are expanding as part of a broader health system reform aligned with Vision 2030. According to the Ministry of Health’s (MoH) standard policy, UCCs are intended to manage patients classified as level 3 under the Canadian Triage and Acuity Scale (CTAS), such as those presenting with moderate dehydration, uncomplicated fractures, or acute abdominal discomfort without red flags [4]. More severe cases (CTAS levels 1 and 2) are directed to EDs, while lower-acuity issues may be addressed in routine primary care clinics. Despite this tiered framework, utilization patterns and public awareness of UCCs remain inconsistent. For example, a 2021 study in Riyadh reported that fewer than one in four patients were aware of urgent care services, although more than half reported using UCCs due to long waiting times or lack of appointment availability in traditional primary healthcare (PHC) centers [5]. Furthermore, the most common presenting complaint in that study was symptoms consistent with the common cold, a condition often managed with symptomatic treatment, but also at risk of inappropriate antibiotic prescribing.

Prescribing practices within urgent care settings, especially for children, has been a growing area of concern and research internationally. A study in the United States (US) analyzing pediatric urgent care visits reported that 42.2% resulted in antibiotic prescriptions, often for conditions such as otitis media or pharyngitis [6]. Also in the US, a study of over 40 million outpatient acute respiratory illness visits between 2011 and 2018 found that the highest rate of antibiotic prescribing was in UCCs, whereas a more recent study found that between 2021 and 2023, there was an approximately 20% increase in antibiotic prescribing per year [7,8].

In the KSA, national initiatives such as the 2018 antibiotic regulation mandate now require formal prescriptions for antibiotics and have imposed tighter controls on their dispensing. These regulations may have influenced the volume and nature of prescribing in urgent care settings, though data on their implementation and effectiveness at the pediatric level remain limited. The objectives of this study are two-fold: First, to examine the utilization of UCCs among the pediatric population and describe prescribing patterns, with a particular focus on antibiotics. Second, to identify predictors of medication prescribing during pediatric urgent care visits. Given the national push for rational prescribing and the increasing reliance on urgent care services for pediatric needs, understanding these patterns is critical for improving clinical governance, enhancing quality of care, and supporting the implementation of targeted interventions in UCCs.

## 2. Materials and Methods

### 2.1. Study Design and Setting

This study utilized a retrospective chart review methodology and was conducted at the Family and Community Medicine Centre affiliated with Imam Abdulrahman Bin Faisal University’s academic medical city in the Eastern Province of the KSA. The center provides comprehensive healthcare services to a population base of over 30,000 individuals, including university staff, students, and their families. Services encompass general family medicine clinics for chronic disease management across all age groups, well-baby and child health clinics, geriatric, diabetes, metabolic, and mental health clinics, women’s health services, and a strong preventive care program covering immunizations, premarital counseling, Hajj and travel medicine, and lifestyle risk screening. The UCC functions as a fast-access track supported by a six-bed treatment and observation area for acute, non-emergent health concerns that require prompt attention. The urgent care pathway is designed to triage and manage cases that fall outside the scope of routine appointments but do not meet ED thresholds. Care in the UCC is delivered by a team of board-certified family physicians and supervised residents, supported by nursing and allied health staff. The UCC is in direct contact with the university hospital’s ED; cases that are unstable are referred there by a dedicated ambulance.

### 2.2. Ethical Considerations

The institutional review board of Imam Abdulrahman Bin University had approved the study with reference number IRB-2024-01-611. The data are kept confidential and were only used for the purposes of research. The study complied with the principles of the Declarations of Helsinki.

### 2.3. Study Participants

The study population included all visits by pediatric patients (<14 years) to the UCC between the 1st of January and the 31st of December 2024. Visits to family medicine clinics or other specialized clinics within the center were excluded.

### 2.4. Data Collection Tool

A list of all pediatric visits to the UCCs during the study period was acquired from the academic medical city’s health information system after ethics approval. A data collection sheet was created to collect the set of variables that will be used in the analysis. These variables were decided on after a review of previous studies and included sociodemographic variables such as age (infants (<1 year old), toddlers (1 year < 3 years), preschoolers (3 years < 6 years), schoolers (6 years < 12 years), and adolescents (12 years < 14 years)), sex, and nationality. Other variables included visit-specific characteristics such as the season of the visit, primary diagnosis, and the type of medications used. The content validity of the tool was reviewed and confirmed by a panel of three experts, comprising a family medicine consultant, a clinical pharmacist, and an epidemiologist. There were no missing data for the variables included in this study, as all fields extracted from the electronic health record were complete.

### 2.5. Data Management and Statistical Analysis

The data were analyzed in STATA software version 15 [9]. Data management included classifying primary diagnoses, which were electronically recorded according to ICD-10, into clinically meaningful categories (e.g., respiratory infections, ENT, gastrointestinal, etc.) after expert review by a family medicine physician and an epidemiologist. This approach was chosen to balance clinical accuracy and epidemiological interpretability. Prescribed medications were categorized into 13 clinically relevant groups based on therapeutic indication and use, after a revision by the clinical pharmacist and family medicine consultant. These categories included vaccines (e.g., hepatitis B, MMR), antibiotics (e.g., amoxicillin, metronidazole), analgesics (e.g., paracetamol, ibuprofen), respiratory inhalers (e.g., salbutamol, fluticasone), systemic antihistamines (e.g., loratadine, diphenhydramine), topical steroids (e.g., hydrocortisone, betamethasone), supplements (e.g., vitamin D, ferrous sulfate), gastrointestinal drugs (e.g., domperidone, polyethylene glycol), neuropsychiatric agents (e.g., methylphenidate, valproate sodium), topical non-steroidal agents (e.g., fusidic acid, miconazole), topical nasal and ENT medications (e.g., xylometazoline, sodium chloride nasal drops), rehydration and electrolyte therapy (e.g., oral rehydration salts, sodium chloride), and diabetes or obesity medications (e.g., insulin, metformin). Seasonality was assessed by grouping the months of visits into seasons: winter (December, January, February), spring (March, April, May), summer (June, July, August), and autumn (September, October, November).

Categorical variables were described as frequencies and percentages. The main outcome was whether a patient was prescribed a medication (yes/no). Bivariate associations were performed through a series of chi-squared tests. Unadjusted and adjusted binary logistic regression analyses were used to compute the odds ratios (ORs) along with their 95% confidence intervals (CIs). Decisions for inclusion of variables in the adjusted model as confounders were based on a directed acyclic graph of relations between variables. The level of significance was set to less than 0.05. Model diagnostics were performed to ensure good model fit, and the model that minimized the AIC and BIC was chosen.

## 3. Results

### 3.1. Sociodemographic Characteristics According to Medication Prescriptions

The total number of pediatric visits to the UCC during the study period was 1016, 62.50% of which involved medication prescriptions. Of the total sample, 50.20% were female patients. Visits from school-aged children were the most common (44.09%), and the least common were for infants (4.82%). A statistically significant association is observed between age and prescriptions, where prescriptions were higher among adolescents and lower among infants (68.09% and 40.82%, respectively). Saudi nationals comprised most visits (70.63%) (Table 1).

### 3.2. Visit Characteristics According to Medication Prescriptions

Table 2 presents the visit characteristics of pediatric urgent care visits according to prescriptions. Most visits occurred during winter, while the least common season of visits was the summer (34.55% and 17.52%, respectively). A highly significant association is found between seasons and prescriptions, with higher proportions of prescriptions during autumn and winter than in spring and summer (*p* < 0.001). With regard to the primary diagnoses, almost 40% of all visits were due to respiratory infections. This was followed by routine check-ups in 25.79% of visits and general fatigue in 7.09%. Medication prescriptions were highest among nutritional, ENT-related, and respiratory infections (100%, 80.49%, and 79.90%, respectively) (*p* < 0.001).

**Figure 1:**
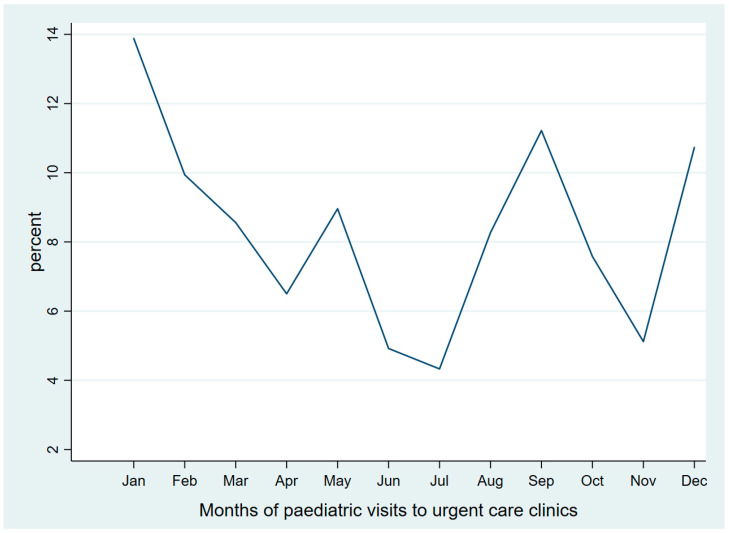
Monthly trends of pediatric urgent care visits in 2024.

Figure 1 presents the temporal trend of urgent care visits across the months of 2024. Notable seasonal variation is seen where the highest proportion of visits was recorded in January, accounting for approximately 14% of the total annual visits. This was followed by a general decline through the subsequent months, reaching the lowest point in July (around 4%). A moderate increase was observed in August, peaking again in September at roughly 11%. After a dip in October and November, the proportion of visits rose sharply in December, exceeding 10%.

**Figure 2:**
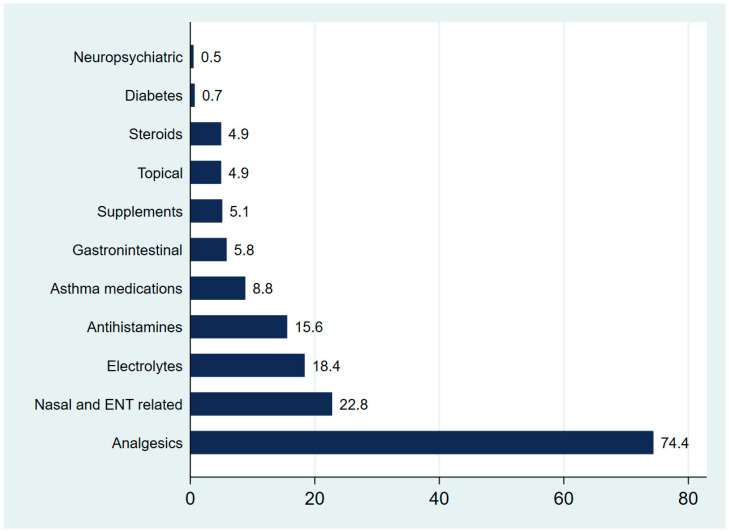
Distribution of non-antibiotic medication prescriptions during pediatric urgent care visits.

Figure 2 displays the distribution of non-antibiotic medications prescribed during urgent care. Analgesics were the most frequently issued, representing 74.4% of all non-antibiotic prescriptions. Nasal and ENT-related medications followed distantly at 22.8%, while electrolytes (18.4%) and antihistamines (15.6%) were also relatively common. The least commonly prescribed medications were neuropsychiatric.

### 3.3. Patterns of Antibiotic Prescriptions by Class

Of the 625 pediatric urgent care visits that had resulted in a medication prescription, 150 were for antibiotics (23.62%). Only 1 patient had 2 antibiotics prescribed (0.67%), resulting in a total of 151 antibiotics prescribed (100.67%). Penicillins were the most prescribed class of antibiotics, accounting for 71.33% of all antibiotics, followed by macrolides (9.33%), nitroimidazoles (8.67%), fluoroquinolones (6.00%), and aminoglycosides (4.00%). Penicillin prescriptions were similarly distributed between males and females, however slight differences were observed for other classes. Regarding age, penicillins were mainly prescribed to preschoolers (83.33%), whereas macrolides and nitroimidazoles were predominantly prescribed to infants (33.33% and 50%, respectively). Also, none of the patients received antibiotics from the cephalosporin, tetracycline, or sulfonamide classes (Table 3).

**Figure 3:**
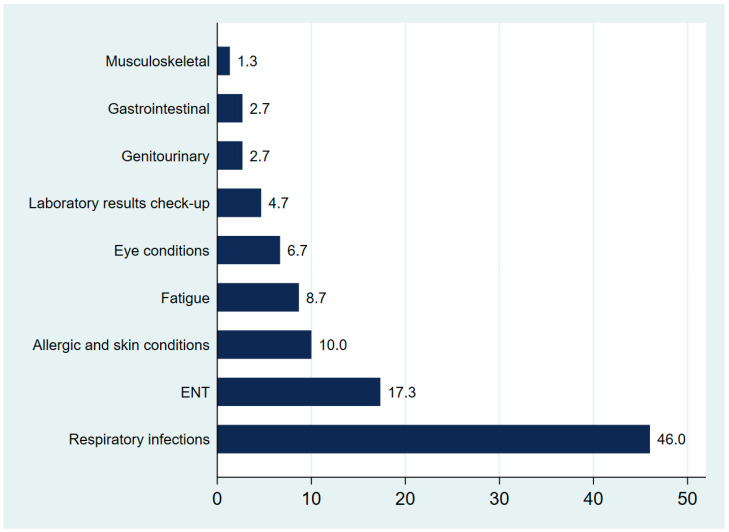
Primary diagnoses associated with antibiotic prescriptions in pediatric urgent care visits.

Figure 3 presents the distribution of diagnoses during pediatric visits that included an antibiotic prescription. It shows that the visits were dominated by respiratory infections, which accounted for 46.0%, followed by ENT-related conditions (17.3%) and allergic or skin complaints (10.0%). Fatigue, eye conditions, and visits for laboratory results were also reported, albeit at lower frequencies.

### 3.4. Multivariable Logistic Regression of Prescriptions in Urgent Care Pediatric Visits

Table 4 presents the unadjusted and adjusted odds ratios of medication prescribing for pediatrics in urgent care. Sex was not a significant predictor of prescriptions. However, for age groups, infants were 70% less likely to have a medication prescription when compared to school-aged children (95% CI = 0.15–0.61), whereas adolescents were 67% more likely to receive a prescription compared to school-aged children (95% CI = 1.04–2.67). Seasonality was significantly associated with prescriptions both before and after adjustment. Both spring and summer were found to be seasons where prescriptions were less likely to occur compared to winter (adjusted OR = 0.52, 95% CI = 0.35–0.77 and adjusted OR = 0.54, 95% CI = 0.35–0.83, respectively). with regard to diagnoses, when compared to respiratory infections, gastrointestinal, neurological, genitourinary, musculoskeletal, general fatigue, and routine check-ups were less likely to receive prescriptions, all of which were statistically significant after adjustment at the 0.05 level.

## 4. Discussion

This study offers a comprehensive epidemiological analysis of pediatric visits to UCCs within a model PHC center in the KSA. To our knowledge, this is among the first studies in the region to focus specifically on pediatric presentations to urgent care within a primary care framework, thereby addressing a critical gap in local health systems research. The findings not only enhance the understanding of service demand and diagnostic trends but also provide valuable insights into prescription patterns, an area of relevance in the context of the KSA’s ongoing healthcare transformation.

The prescribing rate for pediatric patients in the current urgent care setting remains notably high, with 62.5% of visits resulting in a prescription, and 23.6% of these involving antibiotics. This aligns with broader research documenting the frequent use of antibiotics in pediatric care, often beyond clinical guidelines. For example, Nedved et al. (2022) initially found high rates of inappropriate antibiotic use in pediatric UCCs which decreased significantly following targeted stewardship interventions [10]. In EDs, antibiotics were prescribed in 24% of pediatric visits, with a substantial proportion for nonbacterial conditions, further underscoring the issue of overprescription [11,12]. Similarly, in Europe, a study reported that 31.9% of febrile children received antibiotics, many of which were broad-spectrum and not always clinically indicated [13]. However, it is important to note that our study did not assess the appropriateness of prescribing against clinical guidelines, as this was beyond the study objectives.

### 4.1. Visit Characteristics According to Medication Prescriptions

The study on pediatric urgent care visits reveals a notable age-related disparity in prescription rates, with adolescents significantly more likely to receive prescriptions, whereas infants were less likely to receive them when compared to school-aged children. Such patterns have also been seen elsewhere [11,14,15]. This variation may be attributed to differences in illness presentation, clinical judgment regarding necessity of medication, and heightened caution when prescribing to infants due to their increased susceptibility to adverse drug reactions [16].

The seasonal variations observed in pediatric urgent care visits and prescribing practices reflect broader epidemiological patterns influenced by fluctuations in infectious disease prevalence and healthcare-seeking behavior. Peak utilization during the winter months coincides with the seasonal surge in respiratory infections, a well-documented driver of increased healthcare demand in pediatric populations [17,18]. In terms of prescribing, the results show higher proportions of prescribing during winter and autumn, which is also consistent with international studies that report elevated antibiotic use in colder months due to higher infection burdens [19,20]. In contrast, prescriptions were significantly less likely during the warmer seasons, likely reflecting the decreased circulation of respiratory pathogens, lower exposure to school-related transmission, and reduced clinical severity of illnesses during these periods [21]. Similarly, in the US, a 2023 analysis of antibiotic prescriptions found that for respiratory tract diagnoses, prescriptions increased significantly from summer to winter [22]. These findings underscore the influence of seasonality on clinical decision-making and highlight the importance of adaptive healthcare strategies responsive to temporal patterns in pediatric care.

The current study highlights distinct diagnostic patterns influencing medication prescription practices. Respiratory infections were the most frequent causes of urgent care visits and medication prescriptions, which is in line with global data showing acute respiratory tract infection as the most common cause for both antibiotic and symptomatic treatment among children [23,24]. Nearly 80% of children with respiratory infections in this study received medication prescriptions, a finding consistent with concerns raised in other studies about potential overuse of medications in urgent care settings, especially when rapid diagnostic tests are lacking [23,25,26]. ENT-related diagnoses also demonstrated high medication prescription rates, reinforcing findings that upper respiratory tract conditions frequently lead to medication use in pediatric populations [26]. Similarly, the 100% prescription rate for nutritional deficiencies reflects high diagnostic certainty and straightforward therapeutic indications, such as iron or vitamin D supplementation. Studies from Middle Eastern countries highlighted a continued prevalence of nutritional deficiencies among children, which may explain the frequency of supplementation-related prescriptions [27,28].

On the other hand, fewer medications were prescribed for fatigue, neurological problems, and gastrointestinal or musculoskeletal issues. These patterns might indicate a cautious attitude among healthcare providers towards prescribing drugs when there is no clear physical issue, aligning with guidelines for pediatric outpatient care that stress addressing symptoms and advising against antibiotics or medications for non-specific viral infections or mild conditions. This implies that while there could be some areas of potential overprescription, physicians covering urgent care services may be appropriately avoiding unnecessary medication prescriptions for low-risk situations [29,30]. These findings suggest that diagnosis plays a crucial role in prescriber behavior, with respiratory and ENT conditions perceived as more medication-responsive, whereas fatigue, routine care visits, or musculoskeletal pain may prompt more conservative approaches.

With regard to the types of non-antibiotic medications prescribed, analgesics were the most common, highlighting the primary role of symptomatic management in urgent care. Recent studies confirm this trend, identifying analgesics as a mainstay of pediatric urgent care prescribing, often for fever, pain, and upper respiratory complaints [31,32]. Topical nasal and ENT medications were the second most common group of medications. These medications are usually prescribed for cases of upper respiratory symptoms, again reflecting seasonal patterns and symptomatic care priorities. Antihistamines and electrolyte solutions were also common, consistent with their use in allergic rhinitis, viral gastroenteritis, and dehydration, common reasons for pediatric urgent care visits [33,34].

### 4.2. Patterns of Antibiotic Prescriptions by Class

Of the 625 pediatric urgent care visits that resulted in a medication prescription, 23.6% included an antibiotic, and penicillins accounted for most of these prescriptions. This is similar to findings from previous studies in various regions, including Hungary, Jordan, and Ethiopia, where penicillins were consistently the most prescribed antibiotic class in pediatric outpatient settings [35,36,37]. Their widespread use is consistent with current pediatric guidelines, which recommend penicillins as first-line agents for common infections such as pharyngitis, otitis media, and streptococcal tonsillitis.

Interestingly, our study found no prescriptions of cephalosporins, sulfonamides, or tetracyclines. This contrasts with international findings where cephalosporins constitute a substantial portion of pediatric antibiotic prescriptions. For example, in Hungary, broad-spectrum cephalosporins were among the most frequently used classes in outpatient pediatric care [35]. Similarly, in Ethiopia, ceftriaxone was the most prescribed agent, often used as monotherapy or in combination [37]. In contrast, our setting showed no cephalosporin use, likely due to local formulary restrictions. A similar pattern was observed in a local study, where high antibiotic use was reported (66%), but cephalosporin utilization remained limited, likely reflecting formulary and policy constraints [38]. Even within the Gulf region, variation exists, such as in Abu Dhabi, where cefaclor (a second-generation cephalosporin) accounted for 31.1% of prescriptions [39]. These comparisons underscore how national and institutional prescribing policies heavily influence class-specific antibiotic usage.

Age-based analysis within our urgent care population revealed that infants comprised a smaller proportion of total antibiotic prescriptions. This is in accordance with clinical expectations, as infections in this age group are frequently viral and self-limiting. Penicillins were the dominant class across age groups; however, some prescriptions for macrolides and nitroimidazoles in infants were noted, likely topical agents for localized skin or mucosal infections seen commonly in urgent care visits. Sex-based differences in prescribing were minimal in our study, consistent with findings from a clinical audit in Abu Dhabi [39]. However, international studies have reported higher antibiotic use among females [40,41]. Such variability may be influenced by cultural factors, healthcare-seeking behavior, or clinician preferences in urgent care settings.

### 4.3. Implications and Recommendations

Findings from this epidemiological study have significant implications. For epidemiologists, public health and primary care practitioners, the high proportion of urgent care visits resulting in medication prescriptions, particularly among respiratory and ENT-related conditions, reflects areas of high service demand and can inform resource allocation, disease surveillance, health forecasting, health education, and preventive strategies. Second, the observed seasonal variation, with prescriptions peaking in autumn and winter, highlights the influence of infection cycles on urgent care utilization and underscores the need for flexible service planning that adapts to temporal fluctuations in pediatric morbidity, which may include vaccination strategies and other preventive care interventions. Third, the clear age-related differences in prescribing suggest that adolescents and infants represent distinct patient groups with differing clinical management patterns, meriting further exploration in larger multicenter studies.

Although we did not assess the appropriateness of prescribing, the patterns described here provide a foundation for future audits that could link epidemiological trends with guideline adherence. By identifying the diagnoses and seasons most strongly associated with prescribing, this study offers practical signals for where future quality-improvement or stewardship initiatives may be most relevant, pending the availability of key clinical variables such as weight for dose validation. Finally, these insights can support national health transformation efforts under Vision 2030 by strengthening the evidence base for pediatric urgent care services and ensuring that prescribing practices are monitored in a systematic, data-driven manner.

### 4.4. Strengths and Limitations

This study is among the first in the KSA to examine pediatric urgent care prescribing patterns within a model PHC center capturing seasonal variations. The use of standardized electronic records enhances data reliability and internal validity. However, this study has several limitations. First, the retrospective design restricts causal inference, and the findings depend on the accuracy of electronic health records. Misclassification of diagnoses or prescriptions may have occurred due to variability in coding or documentation practices. Although we do acknowledge its significance in clinical practice, the assessment of the appropriateness of prescriptions was beyond the objective of the current study, and further research on this is suggested. Potential biases, including physician decision-making, parental expectations, and clinical uncertainty, may also have influenced prescribing patterns but were not captured in the dataset. Finally, as the study was conducted in a single model PHC center, findings may not be generalizable to other primary care settings, particularly those with different resources or patient populations.

## 5. Conclusions

This study provides insights into the epidemiology of prescribing during pediatric urgent care visits within a model primary healthcare setting in the KSA. By analyzing diagnostic patterns, seasonal trends, and prescribing practices, with a focus on antibiotic use, we identified key age- and diagnosis-related drivers of medication prescriptions. While prescribing rates appeared high, particularly for respiratory and ENT-related conditions, these findings should be interpreted considering the study’s retrospective single-center design. The results suggest potential areas where evidence-based protocols may be relevant, and they may inform rational prescribing strategies and planning for pediatric urgent care services within the broader context of healthcare transformation under Vision 2030.

## Figures and Tables

**Table 1:** Sociodemographic characteristics of pediatric urgent care visits according to medication prescriptions.

Characteristics	N (%) 1016 (100.00)	Medications Prescribed		*p*-Value
		Yes N (%) 635 (62.50)	No N (%) 381 (37.50)	
**Sex**				0.627
Males	506 (49.80)	320 (63.24)	186 (36.76)	
Females	510 (50.20)	315 (61.76)	195 (38.24)	
**Age group**				<0.007
Infants	49 (4.82)	20 (40.82)	29 (59.18)	
Toddlers	115 (11.32)	65 (56.52)	50 (43.48)	
Preschoolers	263 (25.89)	169 (64.26)	94 (35.74)	
Schoolers	448 (44.09)	285 (63.62)	163 (36.38)	
Adolescents	141 (13.88)	96 (68.09)	45 (31.91)	
**Nationality**				0.21
Saudi	710 (69.88)	435 (61.27)	275 (38.73)	
Non-Saudi	306 (30.12)	200 (65.36)	106 (34.64)	

**Table 2:** Characteristics of pediatric urgent care visits according to medication prescriptions.

Characteristics	N (%) 1016 (100.00)	Medications Prescribed		*p*-Value
		Yes N (%) 635 (62.50)	No N (%) 381 (37.50)	
**Season**				<0.001
Winter	351 (34.55)	249 (70.94)	102 (29.06)	
Spring	244 (24.02)	124 (50.82)	120 (49.18)	
Summer	178 (17.52)	89 (50.00)	89 (50.00)	
Autumn	243 (23.92)	173 (71.19)	70 (28.81)	
**Primary diagnosis**				<0.001
Respiratory infections	402 (39.57)	322 (79.90)	81 (20.10)	
Routine check-up	262 (25.79)	76 (29.12)	185 (70.88)	
Unspecified (e.g., general fatigue)	72 (7.09)	48 (66.67)	24 (33.33)	
Allergic and skin conditions	71 (6.99)	52 (73.24)	19 (26.76)	
Gastrointestinal	55 (5.41)	34 (61.82)	21 (38.18)	
Musculoskeletal and orthopedics	45 (4.43)	31 (68.89)	14 (31.11)	
ENT	41 (4.02)	33 (80.49)	8 (19.51)	
Eye conditions	30 (2.95)	21 (70.00)	9 (30.00)	
Genitourinary and puberty-related	21 (2.07)	7 (33.33)	14 (66.67)	
Neurological disorders	7 (0.69)	3 (42.86)	4 (57.14)	
Endocrine disorders and diabetes	4 (0.39)	3 (75.00)	1 (25.00)	
Nutritional deficiencies	4 (0.39)	4 (100.00)	0	
Developmental, psychiatric, and behavioral	2 (0.20)	1 (50.00)	1 (50.00)	

**Table 3:** Distribution of antibiotic classes prescribed by age and sex among pediatric urgent care visits.

Characteristics of Antibiotic Prescribed Patients ^¥‡^	Penicillins N (%) 107 (71.33)	Macrolides N (%) 14 (9.33)	Fluoroquinolones N (%) 9 (6.00)	Aminoglycosides N (%) 6 (4.00)	Nitroimidazoles N (%) 13 (8.67)
**Males (n = 74)**	53 (71.62)	8 (10.81)	6 (8.11)	4 (5.41)	4 (5.41)
**Females (n = 76)**	54 (71.05)	6 (7.89)	3 (3.95)	2 (2.63)	9 (11.84)
**All age groups**					
Infants (n = 6)	1 (16.67)	2 (33.33)	0	0	3 (50.00)
Toddlers (n = 12)	7 (58.33)	2 (16.67)	0	3 (25.00)	0
Preschoolers (n = 42)	35 (83.33)	3 (7.14)	1 (2.38)	2 (4.76)	1 (2.38)
Schoolers (n = 75)	54 (72.00)	7 (9.33)	7 (9.33)	1 (1.33)	7 (9.33)
Adolescents (n = 15)	10 (66.67)	0	1 (6.67)	0	2 (13.33)

^¥^ Only one patient had two prescribed antibiotics. ^‡^ One patient was prescribed lincosamide, and one patient was prescribed folate pathway inhibitors (combined n = 2 (1.34%)). None of the patients were prescribed antibiotics belonging to the cephalosporin, tetracycline, or sulfonamide classes.

**Table 4:** Logistic regression analysis of predictors of medication prescribing during pediatric urgent care visits.

Characteristic	Odds of Prescribing			
	*p*-Value	Unadjusted Odds (95% CI)	*p*-Value	Adjusted Odds (95% CI)
**Sex**				
Males	0.62	1.06 (0.82–1.37)	0.78	1.04 (0.78–1.40)
Females	Ref			
**Age group**				
Infants	0.002	0.39 (0.21–0.71)	0.001	0.30 (0.15–0.61)
Toddlers	0.16	0.74 (0.49–1.12)	0.10	0.66 (0.40–1.08)
Preschoolers	0.86	1.02 (0.74–1.41)	0.06	0.70 (0.48–1.01)
Schoolers	Ref			
Adolescents	0.33	1.22 (0.81–1.82)	0.03	1.67 (1.04–2.67)
**Seasons**				
Winter	Ref			
Spring	<0.001	0.42 (0.30–0.59)	0.001	0.52 (0.35–0.77)
Summer	<0.001	0.40 (0.28–0.59)	0.005	0.54 (0.35–0.83)
Autumn	0.94	1.01 (0.70–1.45)	0.87	0.96 (0.64–1.44)
**Primary diagnosis**				
Respiratory infections	Ref			
Routine check-up	<0.001	0.10 (0.07–0.14)	<0.001	0.10 (0.06–0.14)
Unspecified (e.g., general fatigue)	0.01	0.50 (0.29–0.86)	0.01	0.47 (0.27–0.84)
Allergic and skin conditions	0.20	0.68 (0.38–1.22)	0.25	0.23 (0.38–1.26)
Gastrointestinal	0.003	0.40 (0.22–0.73)	0.009	0.43 (0.23–0.80)
Musculoskeletal and orthopedics	0.09	0.55 (0.28–1.09)	0.04	0.48 (0.23–0.97)
ENT	0.92	1.03 (0.46–2.33)	0.86	1.05 (0.46–2.41)
Eye conditions	0.20	0.58 (0.25–1.33)	0.37	0.67 (0.29–1.58)
Genitourinary and puberty-related	<0.001	0.12 (0.04–0.32)	<0.001	0.12 (0.04–0.32)
Neurological disorders	0.03	0.18 (0.04–0.85)	0.04	0.19 (0.04–0.93)
Endocrine disorders and diabetes	0.80	0.75 (0.07–7.35)	0.62	0.55 (0.05–6.01)
Developmental, psychiatric, and behavioral	0.33	0.25 (0.01–4.06)	0.42	0.31 (0.01–5.27)

## Data Availability

The data presented in this study are available on request from the corresponding author due to restrictions related to institutional policies; access requires prior approval from the Institutional Review Board.

## Abbreviations

The following abbreviations are used in this manuscript:

UCC	Urgent care clinic
ED	Emergency department
KSA	Kingdom of Saudi Arabia
MoH	Ministry of Health
CTAS	Canadian Triage and Acuity Scale
PHC	Primary healthcare
OR	Odds ratio
CI	Confidence interval
